# Reduction in O-GlcNAcylation Mitigates the Severity of Inflammatory Response in Cerulein-Induced Acute Pancreatitis in a Mouse Model

**DOI:** 10.3390/biology11030347

**Published:** 2022-02-22

**Authors:** Mackenzie Moore, Nandini Avula, Alicia Wong, Megan Beetch, Seokwon Jo, Emilyn U. Alejandro

**Affiliations:** 1 Department of Integrative Biology and Physiology, University of Minnesota Medical School, Minneapolis, MN 55455, USA; macmoore@umn.edu (M.M.); avula010@umn.edu (N.A.); beet0013@umn.edu (M.B.); joxxx057@umn.edu (S.J.); 2 Department of Surgery, University of Minnesota Medical School, Minneapolis, MN 55455, USA; 3 Department of Genetics, Cell Biology and Development, University of Minnesota, Minneapolis, MN 55455, USA; wongx615@umn.edu

**Keywords:** O-GlcNAc transferase (OGT), O-GlcNAcylation, pancreas, pancreatitis, acute pancreatitis, cerulein, inflammation

## Abstract

**Simple Summary:**

Acute pancreatitis (AP) is a common disease with significant co-morbidity and increasing global incidence over the past 40 years. Current understanding of molecular underpinnings that facilitates one’s susceptibility to this inflammatory disease is less understood. The protein O-GlcNAc Transferase (OGT) is a cytosolic/nuclear/mitochondrial post-translational glycosylation enzyme that is highly expressed in the pancreas. Here, we propose that OGT, which has been associated with regulating many inflammatory responses such as NFκB signaling, provides the mechanistic link to AP induction and susceptibility. In this study, a mouse model with pancreatic OGT loss was generated and subjected to cerulein, a common pancreatitis inducer. Pancreas OGT-deficient mice exhibited reduced severity of AP, associated with reduced inflammatory markers as well as decreased macrophage population in the pancreas. In conclusion, these data indicate that OGT is a molecular driver that facilitates cerulein-induced AP in vivo.

**Abstract:**

Acute pancreatitis (AP) involves premature trypsinogen activation, which mediates a cascade of pro-inflammatory signaling that causes early stages of pancreatic injury. Activation of the transcription factor κB (NF-κB) and secretion of pro-inflammatory mediators are major events in AP. O-GlcNAc transferase (OGT), a stress-sensitive enzyme, was recently implicated to regulate NF-κB activation and inflammation in AP in vitro. This study aims to determine whether a pancreas-specific transgenic reduction in OGT in a mouse model affects the severity of AP in vivo. Mice with reduced pancreatic OGT (OGT^Panc+/−^) at 8 weeks of age were randomized to cerulein, which induces pancreatitis, or saline injections. AP was confirmed by elevated amylase levels and on histological analysis. The histological scoring demonstrated that OGT^Panc+/−^ mice had decreased severity of AP. Additionally, serum lipase, LDH, and TNF-α in OGT^Panc+/−^ did not significantly increase in response to cerulein treatment as compared to controls, suggesting attenuated AP induction in this model. Our study reveals the effect of reducing pancreatic OGT levels on the severity of pancreatitis, warranting further investigation on the role of OGT in the pathology of AP.

## 1. Introduction

The incidence of acute pancreatitis (AP) has been increasing globally and carries substantial morbidity and mortality as well as increased healthcare utilization and cost [1,2,3]. The prevailing theory for the pathophysiology of AP centers around premature activation of trypsin, the key digestive protease synthesized in acinar cells, which triggers a cascade of inappropriate activation of digestive proteases and, ultimately, pancreatic injury [4,5,6]. Multiple mechanisms contribute to intra-acinar trypsinogen activation and acinar cell death including pathological calcium signaling and organelle dysfunction [7,8,9]. Although pathological trypsinogen activation is a major contributor to acinar cell necrosis in AP, it is not required to induce a local or systemic inflammatory response characteristic of the disease [10,11,12]. Calcium flux, mitochondrial dysfunction, autophagy, ER stress, lysosomal function, and inflammatory signaling are also reportedly involved. Current literature suggests that trypsin-independent activations of proinflammatory transcription factor nuclear factor κB (NFκB) are major events in AP [13,14,15,16].

NFκB proteins consist of a family of dimeric transcription factors that are involved in the regulation of various cellular functions, and the protein pathway is critical in inflammation and immunity. In the absence of stimuli, NFκB is associated with IκB, an inhibitor that prevents nuclear translocation of NFκB. However, in response to stress agents such as reactive oxygen species (ROS) and cytokines, IκB is phosphorylated by IκB kinase (IKK), ubiquitinated, and degraded. This dissociation allows for NFκB nuclear translocation and transcription of proinflammatory target genes such as IL-6 and TNF-α [13,14,15,16,17]. Cerulein, a cholecystokinin analogue that leads to excessive ROS generation and digestive enzyme secretion in the pancreas, is commonly used to generate animal models of human AP. Activation of the NFκB pathway and, consequently, upregulation of various pro-inflammatory markers have been observed as early events in cerulein-induced AP [13]. As such, investigating the mechanisms that modulate this pathway is essential for the development of AP therapies.

It has been found that the NFκb pathway is regulated by various post-translational modifications including phosphorylation, acetylation, and ubiquitination. More recently, studies have shown that O-GlcNAcylation may also be involved. The protein O-GlcNAcylation is a post-translational modification of cytoplasmic, nuclear, and mitochondrial proteins that is catalyzed by the enzyme O-GlcNAc transferase (OGT) and removed by O-GlcNAcase (OGA) [17]. Like phosphorylation, O-GlcNAc modification is rapid, dynamic, and occurs at serine/threonine residue targets [18,19]. The activity of OGT is modulated by glucose availability and general nutrient flux through the hexosamine biosynthetic pathway (HBP). Acute pancreatitis often correlates with hyperglycemia in patients [20,21]. As a nutrient sensor protein, OGT can integrate nutrient availability (i.e., hyperglycemia) and respond to physiological stressors to provide complex regulation of cell signaling, transcription, and translation [22]. OGT is highly enriched in the pancreas and has been shown to regulate inflammatory signaling pathways involved in AP pathogenesis by promoting IκB degradation.

An in vitro study of cerulein-induced AP using the AR42J rat acinar cell line demonstrated that cerulein treatment increased the expression and activity of OGT, as it is a stress-responsive enzyme [13]. This study also found that the inhibition of OGT in vitro decreased NFκB inflammatory signaling and was associated with decreased severity of AP [13]. Although the mechanisms are not fully elucidated, OGT, via O-GlcNAcylation, can directly modify the NF-κB p65 subunit and its upstream activating kinases IKKα in AR42 J cells [13]. In cholangiocarcinoma cell lines KKU-213 and KKU-214, O-GlcNAcylation was also reported to promote the nuclear translocation of NF-κB [23]. Moreover, inhibition of OGT by the small-molecule inhibitor OSMI-1 reduced osteoclast differentiation in vitro and in vivo by disrupting the translocation of NF-κB p65 [24]. However, the role of OGT in AP in vivo models remains uninvestigated.

In the current study, we aimed to determine if a pancreas-specific transgenic reduction in OGT in an in vivo mouse model affects the severity of cerulein-induced AP [25]. Our findings suggest that the partial loss of OGT in pancreatic tissue reduces the severity of AP induced by cerulein, highlighting OGT as a potential molecular target to ameliorate AP in patients.

## 2. Materials and Methods

### 2.1. Mice and In Vivo Glucose Homeostasis Assessment

**Figure 1:**
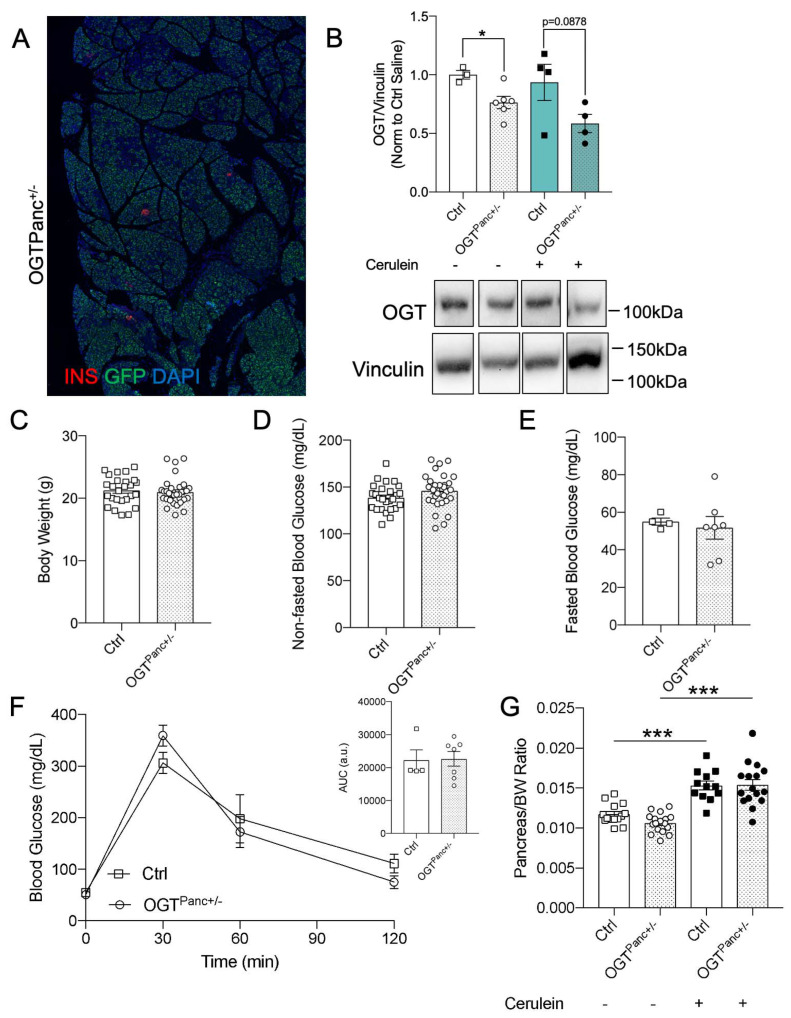
Body weight and glucose homeostasis did not differ between mice with reduced pancreatic OGT and controls. (**A**) Representative immunostaining image of a pancreas from an OGT^Panc+/−^ mouse with Cre reporter GFP, insulin (red), and DAPI (blue). Magnification 10×. (**B**) Western blotting for OGT and Vinculin (loading control) and quantification of OGT over Vinculin, *n* = 3–6. (**C**) Body weight, (**D**) non-fasted, and (**E**) fasted blood glucose of mice before induction of pancreatitis at 8–10 weeks (*n* = 26–33 for (**C**,**D**) and *n* = 4–7 for (**E**)). (**F**) IPGTT and calculated AUC (*n* = 4–7). (**G**) Pancreas to body weight ratio following treatment with saline or cerulein (*n* = 12–17). (**B**–**E**) were presented as mean ± SEM, two-tailed *t*-test, and (**G**) was two-way ANOVA, *p* values, * *p* < 0.05, *** *p* < 0.001.

The following mice were used in the current study: OGT^flox/flox^ mice and Pdx1-cre mice (provided by Dr. Pedro Herrera, University of Geneva, Switzerland) [26]. Mice with reduced pancreatic OGT with the genotype of Pdx1-Cre; OGT^flox/+^ (herein referred to as OGTPanc^+/−^) were generated by breeding OGT^flox/flox^ females with Pdx1-Cre males (Figure 1A). Pdx1-Cre negative sex-matched littermates were used as controls (designated as Ctrl in figures). Due to the nature of Pdx1-cre expression and because OGT is X-linked, thus is susceptible to inactivation, OGT^Panc+/−^ mice have a mosaic expression of recombination [26,27]. Based on previous studies and on Ai6 (CAG-ZsGreen1) Cre-reporter crossed mice (utilizing a CAG system containing ZsGreen1 loxP–flanked GFP protein), a ~40–50% reduction in OGT is expected (Figure 1A) [28]. Experimental animals were genotyped before weaning. All mice were group-housed on a 14:10 light–dark cycle with ad libitum access to a standard chow diet. In a subset of adult mice, intraperitoneal glucose tolerance testing (IPGTT) was performed by fasting the mice for 12 h overnight and measuring fasting blood glucose. A 2 g/kg intraperitoneal injection of a 50% dextrose solution (D50) was then administered. Post-injection blood glucose was collected at 30, 60, and 120 min. All studies were approved by the Institutional Animal Care and Use Committee (#1806–36072A) at the University of Minnesota.

### 2.2. Pancreatitis Induction

At 8 weeks of age, body weight and non-fasted blood glucose were obtained. A blood sample for glucose measurement was obtained from the tail vein with a handheld glucometer. Mice were then randomized to receive hourly intraperitoneal injections of 50 μg/kg cerulein (Sigma-Aldrich, St. Louis, MO, USA, C9026) or an equivalent volume of saline for 8 h, as previously described [29]. Facial vein blood was collected after the completion of the injections. Blood was collected into anticoagulant-coated or microcapillary tubes and centrifuged to obtain serum. This was stored frozen at −80 °C for future biochemical analysis. Mice were then euthanized with CO_2_. Pancreata were freshly harvested, weighed, and divided longitudinally. A portion was preserved in liquid nitrogen for qPCR, and the remainder was fixed overnight in 3.7% formalin and then transferred to 70% ethanol at 4 °C until processing.

### 2.3. Histological Analysis

Pancreatic tissues were embedded in paraffin and sectioned into 5 μm-thick slices through the depth of the pancreas. Hematoxylin and eosin staining were performed as previously described to assess the histological severity of pancreatitis [30]. In brief, this histoscore incorporated the components of edema, inflammatory infiltrate, and necrosis. Ten fields per pancreas were examined and scored at 20× magnification. Scoring was performed by 2 independent researchers that were blinded to the genotype and treatment. Sections for immunofluorescence staining were deparaffinized and underwent antigen retrieval. These sections were then incubated with primary antibodies CD68 (Cellular Signaling Technology, Danvers, MA, USA, 9778S), α-amylase (Sigma-Aldrich, A8273), and insulin (Sigma-Aldrich, I6136) overnight at 4 °C. After washing, sections were incubated with TexasRed- or FITC-conjugated secondary antibodies and DAPI. TUNEL staining was performed using the Sigma-Aldrich Apop Tag Red in situ Apoptosis Detection Kit (Millipore, Burlington, MA, USA, S7165) per the manufacturer-provided protocol with modifications (Sodium citrate buffer with heat was used for the antigen retrieval step). A Nikon Eclipse Ni-E microscope was used to obtain fluorescent images.

### 2.4. Serum Analysis and Western Blotting

Levels of the serum cytokines TNF-α, IL-1β, and IL-6 were measured by the Cytokine Reference Lab at the University of Minnesota (MAGPIX, Luminex, Austin, TX, USA). Amylase (Abcam, ab102523), lipase (Sigma-Aldrich, MAK046), and LDH (Sigma-Aldrich, MAK066) activity assays were performed according to kit instructions. Western blotting was performed in pancreatic lysates as previously described [28]. Antibodies against OGT and Vinculin were purchased from Cell Signaling Technology.

### 2.5. RT-qPCR

TRIzol (Invitrogen) was used to isolate total RNA from pancreas lysates. cDNA was synthesized from total RNA using a High-Capacity cDNA Reverse Transcription Kit (Applied Biosystems, Waltham, MA, USA). Relative gene expression was assessed on an Applied Sciences 7900HT Real-Time PCR System using Power SYBR Green (Applied Biosciences, Salt Lake City, UT, USA), according to the ΔΔCT method, normalized to 36B4 and β-Actin. The primer sequences utilized are listed in (Table S1).

### 2.6. Statistical Analysis

Data are presented as mean ± SEM. Data were analyzed using an unpaired, two-tailed *t*-test for blood glucose and body weight. Repeated measures data for both sexes were analyzed using two-way ANOVA with Sidak multiple comparisons test. The remainder of the data were analyzed using one-way ANOVA with Tukey post hoc test. U-Test was used for TUNEL analysis. Statistical analyses and visualization were performed in GraphPad Prism version 9 with a significance threshold of *p* < 0.05.

## 3. Results

### 3.1. Body Weight, Pancreas Weight, and Non-Fasted Blood Glucose Was Not Affected by Reduced Pancreatic OGT

A full knockout of pancreatic OGT results in pancreas hypoplasia and impaired exocrine and endocrine cell development and function that was not apparent in 8–10 weeks female heterozygous mice under standard diet conditions [26,28]. Given the baseline dysfunction observed in the full pancreatic OGT^Panc−/−^ [26], 8–10 week-old female heterozygotes (OGT^Panc−/+^) were utilized as a haploinsufficiency model to avoid the confounding factors of baseline pancreatic hypoplasia and hyperglycemia. The efficiency of the Pdx1Cre was confirmed by using a GFP-reporter, and as shown in Figure 1A, GFP was expressed partially in the pancreas. We also confirmed OGT protein was reduced in pancreatic lysates of OGT^Panc−/+^ relative to the control mice (Figure 1B). The protein level of OGT was not altered by cerulein treatment. Normal growth and glucose homeostasis were observed in the OGT^Panc+/−^ mice, given that obesity and hyperglycemia are associated with increased severity of pancreatitis [31]. Mice with reduced pancreatic OGT had similar body weight (~20 g), and non-fasted and fasted blood glucose to that of littermate controls (Figure 1C–E). Glucose tolerance testing was performed in a subset of adult mice and did not demonstrate any changes in glucose handling in the OGT^Panc+/−^ mice (Figure 1F). Additionally, pancreas weight was obtained with consideration of saline vs. cerulein treatment, as it was expected that the induction of acute pancreatitis would increase pancreas weight, which was confirmed for both genotypes (controls (Ctrl), *p* ≤ 0.001; OGT^Panc+/−^, *p* ≤ 0.001) (Figure 1G) [32]. Importantly, there was no difference in pancreas weight within the treatment groups.

### 3.2. Mice with Reduced Pancreatic OGT Had Lower Histological Severity of Acute Pancreatitis

**Figure 2:**
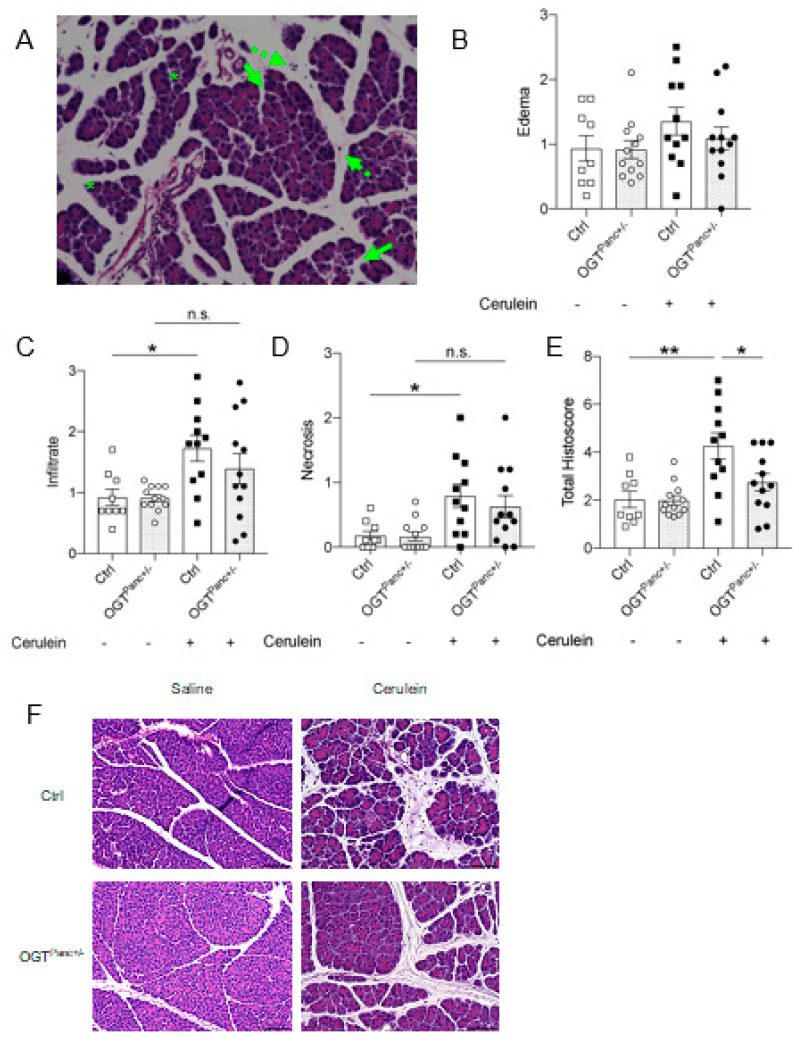
Mice with reduced pancreatic OGT had a decrease in the histological severity of pancreatitis. (**A**) Representative H&E image for scoring severity of pancreatitis of a control mouse treated with cerulein. Edema disrupted and separated acini (solid arrows), inflammatory infiltrate in the parenchyma and between lobules (dashed arrows), focal necrosis (asterisks). Magnification 20×. Histological scoring of (**B**) edema, (C) inflammatory infiltrate, and (**D**) necrosis, respectively, on a scale of 0–3 (*n* = 9–12). (**E**) Cumulative histoscore for the severity of pancreatitis (*n* = 9–12 animals). (**F**) Representative H&E images for each genotype and treatment group. Magnification 20×, scale bar 100 µm. (**B**–**E**) were presented as mean ± SEM, one-way ANOVA. *p* values, * *p* < 0.05, ** *p* < 0.01.

The severity of acute pancreatitis was initially assessed on the histological scoring of edema, inflammatory infiltrate, and necrosis (Figure 2A). Edema was increased with cerulein treatment but did not reach statistical significance (Figure 2B). Inflammatory infiltrate and necrosis were elevated with cerulein treatment in the control mice (Infiltrate: controls, *p ≤* 0.001; Necrosis: controls, *p* = 0.02), but not in OGT^Panc+/−^ mice (Figure 2C,D). Pancreatitis was confirmed with a significant increase in the histoscore of control mice treated with cerulein (*p ≤* 0.001). On cumulative scoring, the severity of pancreatitis was decreased when pancreatic OGT was reduced (*p* = 0.01) (Figure 2E,F).

### 3.3. Lipase and LDH Was Not Elevated with Pancreatitis Induction in Mice with Reduced Pancreatic OGT

**Figure 3:**
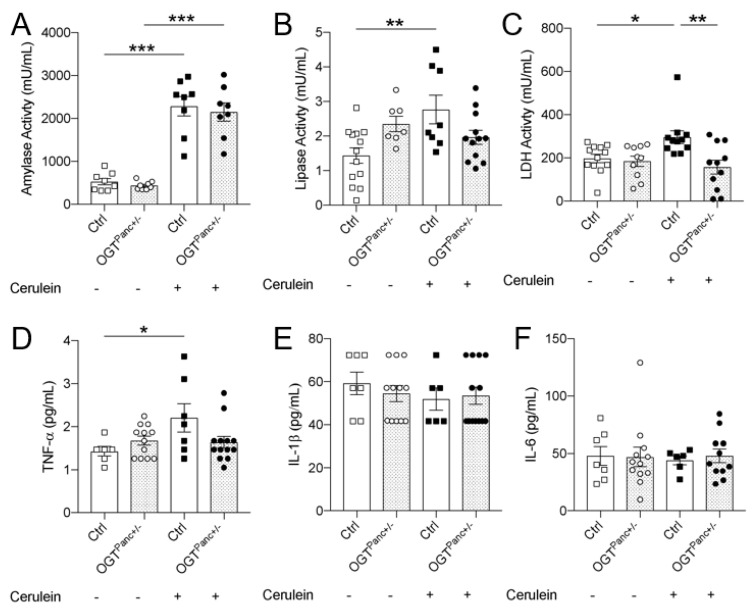
Lipase, LDH, and TNF-α did not increase in OGT^Panc+/−^ mice treated with cerulean. (**A**) Serum amylase (*n* = 8), (**B**) lipase (*n* = 7–13), and (**C**) LDH (*n* = 10–13) as well as (**D**) serum cytokine levels of TNF-α, (**E**) IL-1β, and (**F**) IL-6 (*n* = 6–13) following treatment with saline or cerulein. (**A**–**F**) were presented as mean ± SEM, one-way ANOVA. *p* values, * *p* < 0.05, ** *p* < 0.01, *** *p* < 0.001.

Systemic evidence of pancreatitis was next assessed with a biochemical analysis of serum. Amylase and lipase were obtained to confirm pancreatitis and to assess the degree of pancreatic injury. Amylase was elevated with cerulein treatment to similar levels in controls (*p ≤* 0.001) and OGT^Panc+/−^ (*p ≤* 0.001) (Figure 3A). Lipase levels were also increased with induction of pancreatitis but only in control mice (*p* = 0.004) (Figure 3B). Notably, OGT^Panc+/−^ mice did not demonstrate a significant increase in lipase levels with cerulein treatment compared to saline-treated mice of the same genotype. There was a non-significant trend toward increased lipase in saline-treated OGT^Panc+/−^ mice compared to saline-treated controls (*p* = 0.10). Elevated lactate dehydrogenase (LDH) is associated with tissue injury and, in the context of pancreatitis, is predictive of increased disease severity [33]. Cerulein treatment resulted in a significant increase in LDH in controls (*p* = 0.03) but not in OGT^Panc+/−^ mice (Figure 3C).

### 3.4. OGT^Panc+/−^ Mice Did Not Exhibit an Increase in Serum TNF-α with Induction of Pancreatitis

The systemic inflammatory response was further evaluated by obtaining serum cytokine levels that can be elevated in pancreatitis, particularly when the disease is severe [34]. There was an elevation in serum TNF-α levels with cerulein treatment for controls (*p* = 0.03) but not OGT^Panc+/−^ mice (Figure 3D). Furthermore, when treated with cerulein, mice with reduced pancreatic OGT had a trend toward lower TNF-α levels compared to controls (*p* = 0.08). For IL-1β and IL-6 levels, there were no changes with cerulein treatment amongst controls and mice with reduced pancreatic OGT (Figure 3E,F).

### 3.5. Mice with Reduced Pancreatic OGT Had Similar Levels of Cell Death and without a Significant Increase in Macrophage Infiltrate following Cerulein Treatment

**Figure 4:**
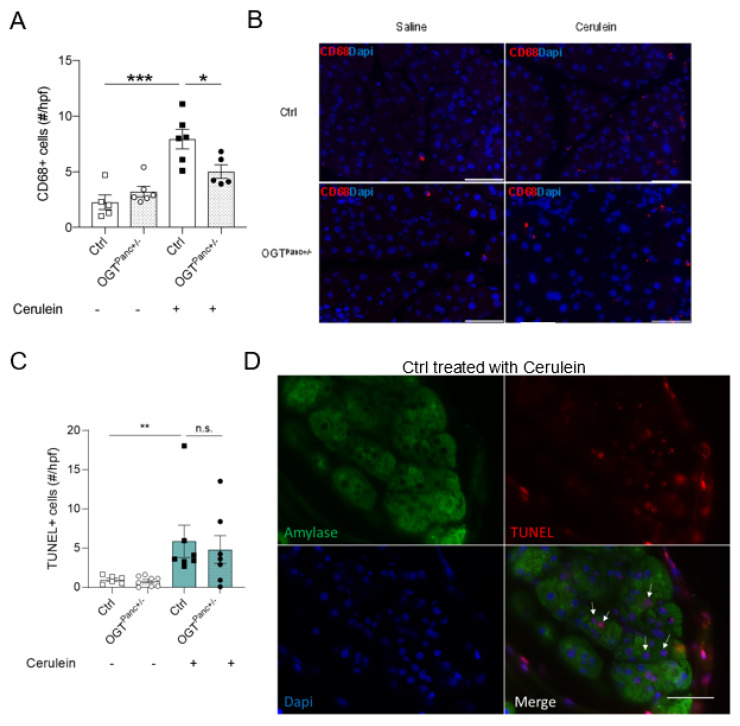
Macrophage infiltrate following cerulein treatment was reduced when pancreatic OGT was reduced. (**A**) Macrophage infiltrate, as determined by CD68+ cells in the pancreatic tissue (*n* = 5–6). (**B**) Representative immunostaining images for each genotype and treatment group with CD68 (red) and DAPI (blue). Magnification 60×, scale bar 50 µm. (**C**) Quantification of TUNEL positive cells/high-power field (*n* = 6–8 animals). (**D**) Representative image from a control treated group with Amylase (green), TUNEL (red), and DAPI (blue). Magnification 60×, scale bar 50 µm. A was presented as mean ± SEM, one-way ANOVA, and C was presented as mean ± SEM, *U*-test. *p* values, * *p* < 0.05, ** *p* < 0.01, *** *p* < 0.001.

We next aimed to quantify and characterize the pancreatic immune infiltrate by identifying macrophages using the CD68 cell marker. There was a marked increase in the inflammatory infiltrate in the control mice that received the cerulein treatment, as indicated by increased macrophage presence (*p ≤* 0.001). However, there was no change between the saline and cerulean treatment in OGT^Panc+/−^ mice (Figure 4A,B). Within the cerulein treatment group, OGT^Panc+/−^ mice had a significantly lower macrophage count in comparison to the control mice (*p* = 0.02). Next, cell death was assessed using a TUNEL assay, which showed a significant increase with induction of pancreatitis by cerulean in the control mice (Figure 4C,D). However, there was a non-significant difference in cell death between the OGT^Panc+/−^ mice and mice treated with saline or cerulein.

### 3.6. Ccl2 mRNA Was Elevated in Cerulein Treated OGTPanc^+/−^ Mice

**Figure 5:**
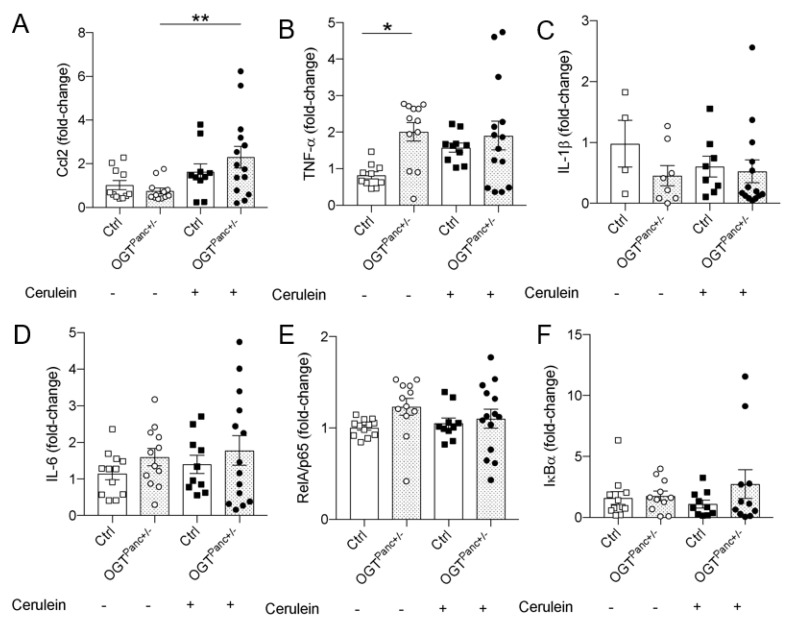
Pancreatic mRNA of the inflammatory and NFκB response. (**A**) Pancreatic mRNA levels of Ccl2, (**B**) TNF-α, (**C**) IL-1β, (**D**) IL-6, (**E**) RelA/p65, and (**F**) IκBα. Values are relative to the saline-treated control condition and normalized to the reference gene 36B4, *n* for experiments = 4–10 animals. (**A**–**F**) were presented as mean ± SEM, one-way ANOVA. *p* values, * *p* < 0.05, ** *p* < 0.01.

Pro-inflammatory mediators are involved in the pathophysiology of AP, and TNF-α, IL-6, IL-1β, and monocyte chemoattractant protein-1 (MCP-1/Ccl2) have been shown elevated in the plasma of patients with AP. To further evaluate the local inflammatory signaling response, mRNA levels of key chemokines and cytokines from pancreatic tissue were assessed. There was a significant increase in Ccl2 mRNA for OGT^Panc+/−^ mice treated with cerulein (*p* = 0.03), which was not evident in controls (Figure 5A). An increase in pancreatic mRNA levels of TNF-α was evident between control and OGT^Panc+/−^ mice at basal (Figure 5B). However, with cerulein treatment, a trend toward increased TNF-α was observed in control mice (Figure 5B). IL-1β and IL-6 mRNA levels were comparable between genotype and treatment groups (Figure 5C,D). Given the importance of the NFκB pathway in the pathophysiology of pancreatitis, mRNA levels for the key transcription factor RelA and the inhibitory protein IκBα were measured. With this approach of assessing the NFκB pathway, there was no difference in the transcription levels of RelA or IκBα (Figure 5E,F).

## 4. Discussion

Hyperglycemia can be both the result and the driver of inflammation and is associated with worse pancreatitis outcomes [35,36]. O-GlcNAcylation increases during times of hyperglycemia and increased nutrient flux through the HBP [37,38]. OGT has been considered to mediate several complications of hyperglycemia such as ER stress and mitochondrial dysfunction [39,40,41]. Thus, the induction of a hyperglycemic state or supplementation of HBP substrates such as glucosamine may potentiate the impact of OGT in the pathophysiology of pancreatitis [42]. Using a genetic model with haploinsufficiency of OGT, the enzyme responsible for O-GlcNAcylation, we observed the attenuation of the severity of acute pancreatitis induced by cerulein. On histological evaluation, OGT^Panc+/−^ mice had decreased severity of pancreatitis. Lipase, LDH, and TNF-α failed to increase with cerulein treatment in mice with reduced pancreatic OGT, indicative of less severe disease development [33]. In addition, the pancreas of cerulein-treated OGT^Panc+/−^ mice had decreased macrophage infiltration in comparison to cerulein-treated control mice. These findings support a previous study by Yang et al., where OGT ablation suppressed macrophage proinflammatory activation and prevented diet-induced metabolic dysfunction in mice [43]. Based on the assessment of components of NFκB and its downstream effectors, there was not a definitive impact on RelA and IκBα mRNA levels in OGT^Panc+/−^ mice as previously postulated [38,44,45,46,47,48,49]. Thus, future studies will investigate the phosphorylation status and localization of key proteins involve in the signaling pathways of NFκB in the pancreas of OGT^Panc+/−^ mice treated with or without cerulein. In addition to NFκB, it would be important in the future to identify other pancreatic proteins O-GlcNAc modified by OGT in response to cerulein treatment.

Activation of the NFκB pathway has been identified as an important component in the propagation of inflammatory signaling during acute pancreatitis [11]. In contrast to studies of chronic inflammation related to metabolic disease, O-GlcNAcylation of NFκB following acute events such as hemorrhage and ischemia have demonstrated anti-inflammatory effects [49,50]. Interestingly, in the setting of acute vascular injury, glucosamine treatment increased O-GlcNAcylation and prevented the phosphorylation of serine 536 on NFκB, which increases its affinity for inhibition by IκB and subsequently inhibited expression of Ccl2 [50,51]. Our study identified an increase in Ccl2 when pancreatitis was induced in mice with reduced pancreatic OGT; this result is consistent with this previous work in the context of vascular injury. In the setting of hemorrhage in rats, increased O-GlcNAc levels by glucosamine treatment improved end-organ perfusion and decreased the serum levels of proinflammatory cytokines including TNF-α and IL-6 [48,49]. Cardiac function was also improved in the glucosamine treatment group, and cardiac tissue was found to have less IκB phosphorylation, and hence NFκB signaling remained inhibited [49]. In contrast, Zhang et al. demonstrated that OGT-mediated O-GlcNAcylation promotes NF-κB signaling activation and inflammation in pancreatic acinar cells, which might promote the progression of pancreatitis [13]. Thus, the effect of O-GlcNAcylation on the NFκB pathway produces gene and stimulus-specific effects that depend on the cellular context and physiological situation [18,38]. It is also important to recognize that glucosamine treatment and genetic manipulation of OGT (affecting both O-GlcNAcylation and non-enzymatic actions) may have different outcomes.

## 5. Conclusions

In conclusion, this study reveals the functional importance of OGT in the induction of cerulein-induced pancreatitis in vivo using female OGT^Panc+/−^ mice. This is consistent with previous in vitro work that demonstrated that decreased OGT reduces the severity of pancreatitis [13], but a significant effect on the genes involved in the NFκB pathway was not identified in the current model. Further exploration of the mechanisms of OGT in the pathology of AP should be conducted in an inducible pancreatic OGT KO model using both adult male and female mice. Finally, future analysis on other components of AP induction such as Ca^2+^ signaling, mitochondrial dysfunction, ER stress, autophagy, and lysosomal function as they relate to OGT and pancreatitis are needed to clarify the impact of O-GcNAc post-translational modification on the progression of this disease state.

## Data Availability

Data available on request.

## Supplementary Materials

The following supporting information can be downloaded at: https://www.mdpi.com/article/10.3390/biology11030347/s1, Figure S1: Pancreatic mRNA of the inflammatory and NFκB response; Table S1: qPCR Primer.
