# Supplementary material for: Reduction in O-GlcNAcylation Mitigates the Severity of Inflammatory Response in Cerulein-Induced Acute Pancreatitis in a Mouse Model
Source: Biology (Basel). 2022 Feb 22;11(3):347. doi: 10.3390/biology11030347 (PMC8945657; doi:10.3390/biology11030347)
Supplement: Supplementary file 1 [file biology-11-00347-s001.zip › biology-1576425-supplementary.pdf]

Fig 1B

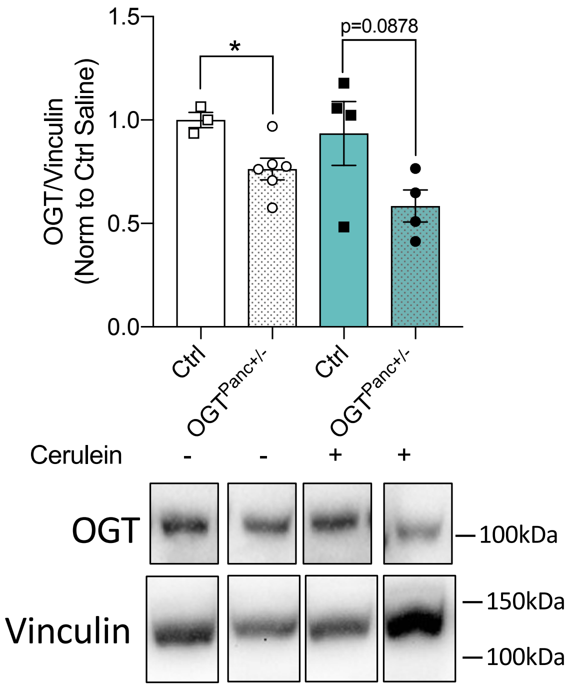

1.5
1.0
0.5
0.0
OGT/Vinculin
(Norm to Ctrl Saline)
*
p=0.0878
Ctrl
OGTPanc+/-
Ctrl
OGTPanc+/-
Cerulein
-
-
+
+
OGT
Vinculin
100kDa
150kDa
100kDa

Raw

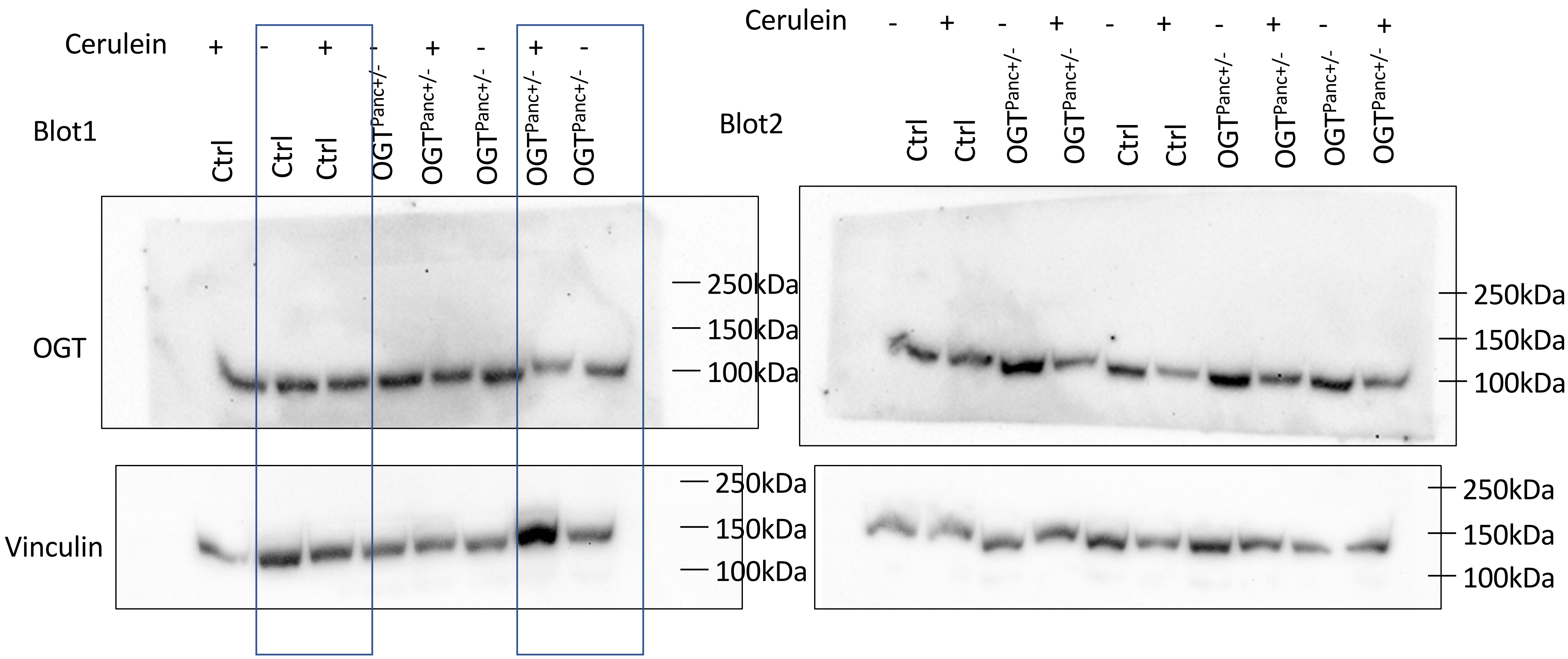

Cerulein
+
-
+
-
+
-
+
-
Blot1
Ctrl
Ctrl
Ctrl
OGTPanc+/-
OGTPanc+/-
OGTPanc+/-
OGTPanc+/-
OGTPanc+/-
OGT
Vinculin
250kDa
150kDa
100kDa
250kDa
150kDa
100kDa
Cerulein
-
+
-
+
-
+
-
+
-
+
Blot2
Ctrl
Ctrl
OGTPanc+/-
OGTPanc+/-
Ctrl
Ctrl
OGTPanc+/-
OGTPanc+/-
OGTPanc+/-
OGTPanc+/-
250kDa
150kDa
100kDa
250kDa
150kDa
100kDa

Quantification

Blot 1						
**Lane**			**OGT Intensity**	**Vinculin intensity**	**OGT/Vinculin**	**Normalized to Ctrl Saline**
**2**	Ctrl	Saline	46.99	86.1	0.545600204	1
				**Avg. WT**	**0.545600204**	
**1**	Ctrl	Cerulein	46.07	71.7	0.642571233	1.177732758
**3**	Ctrl	Cerulein	43.44	75.3	0.576787232	1.057160953
**4**	OgtHET	Saline	35.85	86.2	0.415970482	0.762408955
**6**	OgtHET	Saline	37.97	71.8	0.528759645	0.969133883
**8**	OgtHET	Saline	29.13	68.8	0.423388328	0.776004709
**5**	OgtHET	Cerulein	41.35	61.9	0.667937452	1.224225076
**7**	OgtHET	Cerulein	28.57	127	0.225393895	0.413111823

Blot 2						
**Lane**			**OGT**	**Vinculin**	**OGT/Vinc**	**Norm to Ctrl Saline**
**1**	Ctrl	Saline	42.33	46.9	0.903386901	1.063784072
**5**	Ctrl	Saline	55.55	69.9	0.795053459	0.936215928
				**Avg. WT**	**0.84922018**	
**2**	Ctrl	Cerulein	39.14	45.1	0.868696733	1.022934633
**6**	Ctrl	Cerulein	19.18	46.7	0.410798424	0.48373606
**3**	OgtHET	Saline	42.43	63.5	0.668225771	0.786869868
**7**	OgtHET	Saline	35.88	73.4	0.488655245	0.575416431
**9**	OgtHET	Saline	37.94	63.1	0.601162938	0.707899968
**4**	OgtHET	Cerulein	24.8	57.3	0.432654058	0.509472182
**8**	OgtHET	Cerulein	35.2	54.2	0.64970187	0.765057032
**10**	OgtHET	Cerulein	24.97	45.3	0.55080839	0.648604924
